# Impact of Melatonin on Skeletal Muscle and Exercise

**DOI:** 10.3390/cells9020288

**Published:** 2020-01-24

**Authors:** Alessandra Stacchiotti, Gaia Favero, Luigi Fabrizio Rodella

**Affiliations:** 1 Anatomy and Physiopathology Division, Department of Clinical and Experimental Sciences, University of Brescia, Viale Europa 11, 25123 Brescia, Italy; gaia.favero@unibs.it (G.F.); luigi.rodella@unibs.it (L.F.R.); 2 Interdepartmental University Center of Research “Adaptation and Regeneration of Tissues and Organs (ARTO)”, University of Brescia, 25123 Brescia, Italy

**Keywords:** melatonin, sarcopenia, fibromyalgia, physical training, mitochondria, mitophagy, aging, rodents

## Abstract

Skeletal muscle disorders are dramatically increasing with human aging with enormous sanitary costs and impact on the quality of life. Preventive and therapeutic tools to limit onset and progression of muscle frailty include nutrition and physical training. Melatonin, the indole produced at nighttime in pineal and extra-pineal sites in mammalians, has recognized anti-aging, anti-inflammatory, and anti-oxidant properties. Mitochondria are the favorite target of melatonin, which maintains them efficiently, scavenging free radicals and reducing oxidative damage. Here, we discuss the most recent evidence of dietary melatonin efficacy in age-related skeletal muscle disorders in cellular, preclinical, and clinical studies. Furthermore, we analyze the emerging impact of melatonin on physical activity. Finally, we consider the newest evidence of the gut–muscle axis and the influence of exercise and probably melatonin on the microbiota. In our opinion, this review reinforces the relevance of melatonin as a safe nutraceutical that limits skeletal muscle frailty and prolongs physical performance.

## 1. Skeletal Muscle Structure and Function in Aging and Diseases 

Skeletal muscular tissue is the most represented tissue in the human body and is essential for voluntary movements and postural maintenance [1,2]. However, it has additional important roles such as thermal regulation, nutritional balance, glucose uptake, and endocrine activity [3,4]. 

Before understanding the progressive changes of skeletal muscle induced by aging and related diseases, it is necessary to focus on its structure and ultrastructure.

In adult mammals, skeletal muscle is composed by different cell types—multinucleated myofibers, or myotubes, and satellite cells—beneath the sarcolemma, able to regenerate surrounded by the epimysium [5,6]. Myofibers are then collected in bundles surrounded by the perimysium layer. Finally, many bundles in different spatial orientation constitute the gross muscle mass fixed with a tendon to the skeleton [7]. Moreover, fibroblasts, adipocytes, vessels, and neuromuscular junctions complete the complex muscle anatomy [8,9,10,11].

Remarkably, a single myofiber is a post-mitotic highly differentiated cell containing multiple peripheral nuclei, a set of contractile myofilaments, the sarcoplasmic reticulum for calcium flux, and mitochondria for providing energy for movement [12]. Ultramicroscopic studies have characterized, in human and rodent muscle, different mitochondria subtypes called sub-sarcolemmal (SSM), perinuclear, and inter-myofibrillar (IMF) mitochondria [13,14,15]. These definitions indicate the peculiar localization within the skeletal myotubes, even if different mitochondria may also have biochemical and proteomic specializations. Indeed, SSM mitochondria are involved in gene transcription and resistance to reactive oxygen species (ROS), while IMF mitochondria are devoted to oxidative phosphorylation, ATP production, and directly drive calcium (Ca^2+^) ions flux in calcium release units (CRUs) or triads [16]. Remarkably, a recent tridimensional reconstruction of mitochondria in skeletal muscles identified novel subtypes of mitochondria associated with vessels, called para-vascular mitochondria, all interconnected in the sarcomere [17]. Therefore, human skeletal muscle mitochondria, assessed by the mitochondrial complexity index (MCI), are dynamic within each myofiber, even if they are mainly present at the Z-line [18]. It is important to state that mitochondrial connectivity and branching in muscle depend on the mitochondrial DNA (mtDNA) and might change according to the aerobic oxidative metabolism [19,20]. 

Indeed, skeletal muscle fibers exist into two different types according to isoforms of structural proteins called myosin heavy chain (MYH) and tropomyosin [21]. The most common are type I or slow-twitch myofibers, and type II, or fast-twitch myofibers. This last type is further divided into type II A and type II X [22,23]. However, considering the energy consumption and the ATP production, slow types II A and I fibers rely upon an aerobic oxidative metabolism and constitute the red muscles. On the contrary, fast type II B fibers adopt glycolysis and make up the white muscles [24,25]. Intriguingly, in anaerobic glycolytic fibers, mitochondria are associated with the sarcomere I-band, while in oxidative fibers, mitochondria are numerous in I-band and A-band [26]. Remarkably, in fast-twitch myofibers in red muscles, all triads are associated with mitochondria, and their tether causes Ca^2+^ ions release from sarcoplasmic reticulum and ATP production [27,28]. Therefore, size, activity, and adaptability of mammalian skeletal muscles to movement largely depend on the size and the type of individual fibers and their mutual transition and plasticity [29,30,31]. However, metabolic requirements deeply affect mitochondria shape and dynamic in skeletal muscles [32]. A balance between short and elongated fused mitochondria is necessary and is linked to “fusion” and “fission” processes and relative shaping proteins [33,34]. All these morphological changes are critical for the respiratory activity and for driving proper mitophagy, i.e., the process of dysfunctional mitochondria cleaning in muscles [35]. 

Aging inevitably affects skeletal muscle structure and function in mammalians [36,37,38,39]. Both quality and strength of muscle fibers progressively change in the elderly, affecting mobility and independence. Altered excitation–contraction coupling together with abnormal calcium flux and sarcoplasmic reticulum organization in myofibers induce weakness and loss of intrinsic force in old muscles [40,41,42].

The most common indicator of skeletal muscle aging is sarcopenia. Sarcopenia, i.e., the qualitative and the quantitative reduction of muscle mass and strength, is due to irreversible reduction in type II A oxidative red fibers. Recently, the European Working Group on Sarcopenia in Older People (EWGSOP) published a revised definition of sarcopenia associated with proper diagnosis and management [43]. However, these adverse events affect individuals starting in the fourth decade of their life and get worse by sedentary lifestyle [44,45]. Therefore, sarcopenia is a progressive multifactorial process linked to structural, biochemical, and metabolic dysfunctions [46]. 

From a morphological point of view, aged skeletal muscles dramatically lack satellite cells, regular capillary blood flow, well-organized triads, and calcium entry [47,48,49]. Intriguingly, aged muscles present central nuclei and fill with cells such as adipocytes, inflammatory cells, and fibroblasts intensely producing collagen [50,51,52]. A recent study in sarcopenic-aged patients compared with non-sarcopenic controls reported a peculiar inflammatory profile, called “cytokinome”, mainly characterized by higher C-reactive protein in men versus women [53]. Remarkably, in aged muscle, mitochondria greatly change structure and function as reported by recent authoritative reviews [32,54]. Currently, the recovery of abnormal mitophagy is a new and promising target for treating sarcopenia [55]. Another extreme consequence of muscle frailty in aging is disuse atrophy, which dramatically affects mitochondrial homeostasis. 

The main evidence of muscle atrophy is the loss of strength due to enhanced muscle protein degradation and the progressive loss of type I and II A oxidative fibers [56,57]. In atrophying muscle, mitochondria biogenesis is affected, and molecular pathways involved in mitochondria maintenance are disrupted already after few weeks of immobilization [58]. Furthermore, mitophagy and fusion-fission events are completely dysregulated [59]. Indeed, mitochondria are short, fragmented, and particularly prone to excessive mitophagy that progressively causes a deficit of their number and energy supply in atrophying muscle [60]. Consequently, loss of ATP production in muscle induces ROS contributing to inflammation, mtDNA deletion, and apoptosis [61]. The coexistence of dysfunctional mitochondria and ROS in muscle activates the “inflammasome”, an assembly of proteins that activated cytokines or inflammatory signals driven by nuclear factor k-B signaling [62,63]. 

Emerging evidence has related mitochondria biogenesis and activity to peroxisome proliferative activated receptor gamma coactivator 1 alpha (PGC-1α) and to the transcription of downstream genes such as nuclear respiratory factor 2 (Nrf2) and mitochondrial transcription factor (TFAM) [64]. A recent study demonstrated that, in old mice overexpressing PGC-1α, there was less mitophagy due to more effective healthy mitochondria and improved oxidative metabolism in the *tibialis anterior* muscle [65]. Remarkably, Nrf2 deficiency in knockout aged rat induced muscle mass reduction, abnormal mitochondrial dynamics, and biogenesis [66]. 

Other age-related skeletal muscle diseases are crush injury, a common consequence of fall, chronic fibromyalgia, and altered microvascular perfusion. 

In traumatic injury, the age of patients is important because it dramatically influences the regeneration and the repair of skeletal muscle fibers [67]. To restore proper muscle organization after a crush, multiple local and systemic mechanisms such as inflammation, polarization of macrophages, remodeling of fibroblasts, and restoration of neuromuscular junction activity are involved [3]. 

Chronic neuromuscular pain and fatigue, defined as a poor response to sustained muscle tension, are common indicators of fibromyalgia [68]. The complex and still obscure pathogenesis of fibromyalgia includes inflammation, oxidative stress, ROS production, and mitochondrial damage [69]. In particular, the disruption of mitochondrial permeability transition, excessive mitochondrial fusion, and inflammation are markers of the disease [70]. 

Ischemic diseases are largely associated with traumatic muscle crush or thromboembolic events and, if untreated, may lead to irreversible necrosis [71]. Early reperfusion, i.e., the restoration of circulatory flow, is the primary goal to treat muscle ischemia. Unfortunately, reperfusion may induce severe injury in glycolytic muscles due to the local production of ROS in the post-ischemic phase [72]. Dramatic progressive muscle weakness due to the loss of cytoskeletal dystrophin is an adverse genetic condition called Duchenne muscle dystrophy (DMD) reproduced in mdx mice [73]. Even if DMD onset occurs generally at a young age, it resembles the muscular frailty of the elderly. ROS and abnormal calcium homeostasis, largely affecting sarcolemma, concur with muscle degeneration and strong oxidative damage. Despite promising genetic and pharmacological interventions able to attenuate DMD induced changes such as inflammation and vasoconstriction, an effective therapy is still lacking [74]. Main features in adulthood and aging-induced muscle disorders are collected in Table 1.

## 2. Melatonin Alleviates Skeletal Muscle Disorders In Vitro and In Vivo

Melatonin (N-acetyl-5-methoxytryptamine) is an evolutionary-conserved molecule originally isolated from the pineal gland and considered a regulator of circadian rhythms and seasonal breeding [75,76]. Actually, melatonin has multiple extraordinary functions such as anti-tumor, antioxidant, and anti-inflammatory indolamine [77,78,79]. In mammals, melatonin has been identified in all body fluids and in several extra-pineal sites such as skin, gastrointestinal tract, liver, kidney, immune system, testis, and skeletal muscles [80]. Remarkably, during the last decade, the indole has been detected in several edible plants, eggs, and fish, assuming an interesting and promising role as a nutraceutical [81,82]. The compelling evidence of decreased endogenous melatonin in senescence triggered intense research on its potential role as a dietary supplement to prevent and treat aging and age-related diseases [83,84]. 

Intriguingly, in postmenopausal women, the drop of urinary melatonin correlated with sarcopenia and, in castrated male rats, melatonin supply slowed muscle atrophy, acting as testosterone [85,86]. Chronic melatonin intake prevented age-related mitochondrial damage in the heart and the diaphragm muscle of accelerated aged SAMP 8-mice [87]. Muscle strength depends on constant regular glucose and insulin levels in the blood, but in sarcopenia, insulin-resistance occurred [88]. Furthermore, inflammation and lower glycolytic potential, assessed as lactate amount, strongly influenced skeletal muscle metabolism in sarcopenia [89]. In the *gastrocnemius* muscle of aged mice altered autophagy, nuclear fragmentation and abnormal lactate production were detected, but all these adverse changes decreased by oral melatonin intake [90]. Remarkably, melatonin supplementation in NLRP3 KO mice was particularly beneficial to retard the onset of sarcopenia in gastrocnemius muscle in aged animals [91]. Interestingly, exogenous melatonin regulated insulin resistance, ameliorated mitochondrial function in rat muscles, and prevented chemically induced apoptosis and endoplasmic reticulum stress in different skeletal muscle cells in vitro [92,93,94,95,96,97]. 

Coto-Montes and co-workers reviewed the promising utility of safe melatonin dietary intake in sarcopenia, even if its therapeutic potential in patients is still controversial [98,99]. Recent evidence in different sarcopenic mice models obtained by single or double KO (DKO) of mitochondrial shaping proteins (DRP1-KO and Opa1-Drp1 DKO) definitively indicated how, in skeletal muscles mitohormesis, the mitochondrial size balance was essential [100,101]. 

Considering the post-mitotic nature of skeletal myofibers, skeletal muscle healing and recovery after prolonged ischemia are other relevant clinical issues [102]. Several studies demonstrated that melatonin attenuated ischemic damage and restored microvascular structure and perfusion in rat cremaster and *gracilis* muscles during ischemia/reperfusion [103,104]. 

Moreover, muscular traumas, often during sports performance, are very common evidence associated with high medical expenses and disabilities. In addition to promising clinical trials with progenitor cells in humans [105], there are convincing data on the beneficial role of melatonin in crushed injured muscles in rodents. Chronic melatonin intake reduced apoptosis, increased twitch force, and accelerated regeneration enhancing satellite cells in muscle injury in mice and in rat [106,107]. Recently, in an experimental compression model of *quadriceps* muscle in rats, melatonin administered two hours after the beginning of the compression and during the following six days improved redox balance and reduced inflammatory markers and tissue damage [108]. 

Chronic muscular pain, cognitive dysfunctions, and sleep disorders are all hallmarks of fibromyalgia linked to reduced urinary secretion of melatonin in women [109]. Considering that an effective therapy to alleviate pain and muscle damage in this syndrome is still lacking, our group studied the effects of melatonin in fibromyalgia in rats. Fibromyalgia was induced by reserpine injection and melatonin administered in tap water, concurrent with reserpine, for one or two months. In rat *gastrocnemius* muscle, melatonin reduced oxidative changes and ameliorated mitochondria shape and cristae, improving voluntary motor activity [110]. More recently, we adopted the same model focusing on mitochondrial markers in rat *gastrocnemius* muscle. Interestingly, PGC1-alpha pathway and mitofusin 2 (MF2), essential indicators of mitochondrial activity and fusion, were affected in reserpine injected rat but preserved after oral melatonin intake [111]. These data strongly indicated that, in the muscle, melatonin directly accumulated in the mitochondria where it was able to sustain proper size and function. 

Altered oxidative balance and abnormal mitochondria characterize DMD, a severe genetic disorder associated with muscle weaning and atrophy [112]. Melatonin, which was successfully administered as a nutraceutical compound in preclinical mice models and in DMD patients, ameliorated muscle metabolism and strength [113,114]. Indeed, the indole sustained the antioxidant muscular potential, increasing total glutathione content and promoting an effective contraction. 

**Figure 1:**
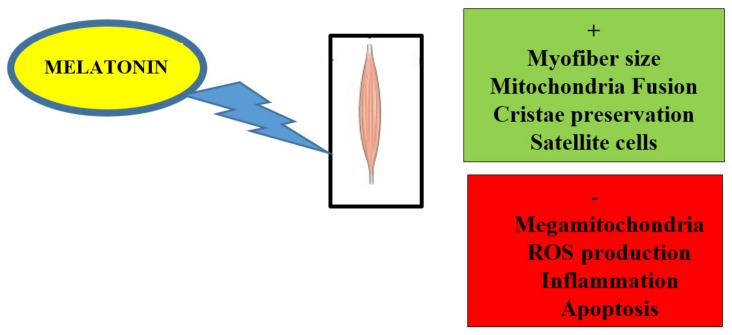
Scheme illustrating improvement (+/ in green) and block (−/ in red) of mitochondrial or muscular events induced by dietary melatonin in aged or damaged skeletal muscle.

A summary of beneficial or promising actions of melatonin in aged induced skeletal muscle diseases can be found in Figure 1.

## 3. Exercise—an Anti-Aging Strategy that Preserves Mitochondria in Skeletal Muscle

Physical activity and proper nutrition represent the best lifestyle measures to prevent and to retard age-related sarcopenia and progressive muscular weakness [115,116]. Remarkably, regular and controlled exercise with advancing age prolongs lifespan and improves skeletal muscle mass and performance [117,118]. 

However, there are different types of physical training with different impact on skeletal muscle composition and metabolism: acute, chronic, or glycolytic and aerobic exercise [44,119]. Different types of exercise can be combined in a mixed training. Exercise greatly remodels skeletal muscle mitochondria size and number and accelerates mitophagy, the peculiar dismantling of damaged mitochondria [120]. Indeed, the proper balance between new synthesis of mitochondria and mitochondrial degradation is dramatically altered in aged skeletal muscles [121]. 

Several studies reported that different muscles aged differently depending on their fiber composition and metabolism. Generally, mitochondrial respiratory activity is well preserved in slow oxidative muscles rich in type I, IIA, and 2X fibers, but damaged in aged fast-twitch glycolytic muscles rich in type IIB fibers [122,123,124]. Recently, Crupi et al. measured the respiratory activity in isolated mitochondria from fast glycolytic *tibialis anterior* muscle versus slow oxidative *soleus* muscle in aged mice and demonstrated that oxidative fibers are preserved while the glycolytic ones are damaged [125]. Thus, a fundamental strategy of physical training in the elderly is to strengthen the oxidative muscles. 

At a cellular level, to alleviate sarcopenia is necessary to improve mitochondria number and turnover of contractile proteins and anti-oxidant enzymes [126]. A proper mitochondrial turnover is crucial in muscle adaptation to exercise given that abnormal proteostasis leads to loss of contractile proteins in aged muscles [127,128]. However, despite controlled physical activity, complete muscle restoration is impossible due to unavoidable oxidative deterioration of fast glycolytic fibers and enhanced expression of age-related genes insensitive to exercise benefit [129,130]. 

Remarkably, there is a strict connection between genotype and phenotype in gene polymorphism for structural proteins and angiotensin-converting enzyme (ACE) [131]. This connection is particularly evident in differences between endurance or power muscles in athletes influencing sports performance, degree of vascularization, and mitochondria1 function [132]. A great deal of evidence indicates that mitophagy in muscle is activated by exercise but is abnormal in aging if mitophagy flux is excessive and mitochondrial quality reduced [133,134]. Multiple receptors and signals are involved to drive proper mitochondria turnover in skeletal muscle and to regulate lysosomal homeostasis. One of the most studied transcription factors activated during exercise and able to regulate mitophagy is transcription factor EB (TFEB) [135]. This molecule is active when dephosphorylated by calcium ions released from the sarcoplasmic reticulum during acute exercise and, after translocation into the nucleus, controls the expression of several genes driving the autophagy–mitophagy steps [136]. Another master regulator of mitophagy in muscle is PGC1α, normally activated during acute exercise [137,138]. Recent study in mice indicated that chronic exercise and stimulated contractile activity ameliorated mitochondria, thus mitophagy was not necessary [139]. However, the use of colchicine as a microtubule polymerization inhibitor allowed measuring the enhanced mitophagy flux in muscles of voluntary wheel trained mice [132]. 

Remarkably, not only mitochondria but also lysosomes are crucial for an efficient mitophagy and the maintenance of mitohormesis. Recently, Triolo and Hood reviewed the beneficial effect of acute and chronic exercise in lysosomal biogenesis and reported that exercise might be a peculiar non-pharmacological “therapy” to clear disrupted cellular components [140]. In particular, the authors stressed that, after physical training in rodent models of lysosomal diseases such as Pompe disease and Danon syndrome, muscle mass and strength increased, while mitophagy and lysosomes production ameliorated. Moreover, the therapeutic potentiality of aerobic and resistance exercise to promote skeletal muscle performance was reported in patients affected by Pompe disease [141,142]. 

Emerging evidence indicates that a progressive resistance program better than acute oxidative exercise is crucial to preserve mobility in aged patients, thus retarding the onset of frailty and related metabolic and cardiovascular diseases [143]. Another beneficial role of exercise is the secretion of muscular cytokines, called myokines, which regulate multiple biological functions [144]. 

Indeed, throughout the blood circulation, myokines influence muscles but also external sites such as the adipose organ [145]. Among over three thousand myokine, it is necessary here to outline fibroblast growth factor 21 (FGF21), a hormone-like molecule mainly secreted by the glycolytic fibers, irisin secreted by the oxidative ones during contraction, and brain-derived neurotrophic factor (BDNF) activated by aerobic exercise [146,147,148]. Remarkably, resistance training is able to recover the secretory activity of aged muscles [149,150]. 

A recent field of research is the analysis of circulating exosomes, a sort of nanovescicles that deliver myokines during exercise from muscles to adipose organs or other sites [151]. Interestingly, new soluble factors delivered by exosomes might represent therapeutic or diagnostic targets of muscle wasting or walking speed decline in aging [152,153]. 

## 4. Impact of Melatonin on Skeletal Muscle Activity and Exercise

Day/night cycles and seasonal rhythms are evolutionary-conserved activities that deeply influence skeletal muscle mass, performance, and mitochondrial function [154,155]. The circadian clock conditions whole body homeostasis and mainly the sleep–wake cycles that are essential for mental and physical fitness [156]. 

In humans, the master regulator of biological rhythms is the suprachiasmatic nucleus (SCN) located in the anterior hypothalamus in the brain [157]. This central area is functionally linked to peripheral sites by factors called Z*eitgebers* in German, or “synchronizers” in English, such as daylight exposure, physical activity, sleep, and eating time habit [158,159]. Remarkably, the SCN pathway linked to retinal ganglion cells produces melatonin, an endogenous Z*eitgeber* able to control vital rhythms during the nighttime, to reduce mitochondrial dysfunctions and chronodisruption [160,161]. In particular, melatonin added in vitro to brain slices stimulated SCN phase via melatonin type-2 receptors and protein kinase C activation [162]. 

Molecular mechanisms regulating circadian rhythms, clock genes, and transcriptional regulators were firstly characterized in *Drosophila* and then extended to higher organisms [163]. Compelling evidence indicates that there is another peripheral clock in skeletal muscle crucial for the maintenance of mitochondrial balance, muscle metabolism, and energy in sarcopenia [164,165,166,167]. 

A recent study demonstrated that old mice fed an obesogenic diet supplemented with nobiletin, a polyphenol agonist of muscle circadian regulator retinoid acid receptor-related orphan receptor (ROR), presented an enhancement of mitochondria activity, energy expenditure, and endurance exercise in the calf muscles, *gastrocnemius* and *soleus* [168]. Moreover, actually single fiber proteomic allows an unbiased determination of full muscle and mitochondrial proteins that are characteristic of a specific myofiber to best correlate its status in health, aging, or metabolic diseases [169]. 

Exercise is another essential nonphotic Z*eitgeber* that synchronizes the circadian pathway and sleep depth and controls muscle physiology during all lifespans but greatly in aging [170,171]. Notably, the direct influence of exercise on the endogenous melatonin secretion is still controversial, probably due to different melatonin estimation in saliva or serum. Indeed, salivary melatonin evaluated in men in the late evening was inversely related to the time of physical activity because it was higher in the morning session versus a late afternoon session of steady-state running, thus the morning fitness may predispose one to regular sleep [172]. Accumulating evidence in rodents indicates that endogenous melatonin and circadian systems are modulated by repeated vigorous exercise able to maintain the synchronous phase. Conversely, Escames et al. reported that, in humans, due to a lack of control on competing *Zeitgebers* and the difficulty to directly estimate the SCN input, the effects of exercise on endogenous melatonin are controversial [173]. Moreover, enhanced urinary melatonin was associated with more grip and *quadriceps* muscle strength in the elderly population [174]. However, in previously sedentary men and women aged 40-75 years after one year of moderate exercise, the urinary melatonin metabolite levels were unchanged [175]. Recently, as a result of a moderate exercise in a hypoxic status (equivalent to at 4500 m altitude), serum melatonin increased, probably to protect against oxygen deprivation [176]. 

In any case, exogenous melatonin is useful as an antioxidant and an anti-inflammatory nutrient for prolonging muscle strength and adaptation during strenuous exercise in rodents and men in adulthood and aging [177,178,179,180]. Melatonin intake before and during exercise reduces glucose resistance and ameliorates antioxidant status in various situations, such as during preparatory training, in a soccer training camp, in resistance, or in high-trained athletes [181,182,183,184]. In particular, during strenuous training and muscular trauma induced in rodents, melatonin supplementation via different routes improved muscle recovery, inhibiting NF-kB activation and inflammatory cytokines and downregulating atrophy pathways [185]. However, Beck et al. reported an ergogenic role of intraperitoneal melatonin in *gastrocnemius* muscle in rat swimming that mimicked long-duration aerobic exercise in human but increased inflammation, probably due to excessive physical performance extension [186,187]. Remarkably, in humans, melatonin was devoid of any side effect despite administration via several routes [188]. Melatonin induced drowsiness must be considered, and the best time to assume the indole is during post-exercise recovery or convalescence [189].

On the contrary, the influence of exogenous melatonin on the physical performance is still debated and controversial. In a recent systematic review, Lopez-Flores et al. indicated that the intake of melatonin might be effective or ineffective depending on the type of physical activity [190]. Indeed, melatonin secretion is limited during aerobic exercise but is enhanced during high-intensity exercise, thus the effects of exogenous melatonin supply are different.

Several studies agreed on the beneficial role of oral melatonin intake to readjust sleep cycles and jet-lag adverse effects after transcontinental flights, making athletes more prone to optimal performance [191]. A single “pharmacological“ dose of melatonin (3 mg) should be taken in daytime to shift the circadian rhythm in the proper direction and to best adapt to the new time zone according to westward or eastward travel. This suggestion might be very useful, for example, for athletes participating to the next 2020 Olympic Games in Tokyo, Japan. Moreover, 10 mg melatonin taken after strenuous exercise in late evening was effective to prolong sleep and to ameliorate short–term activity in the following morning in teenager athletes [192].

Another interesting property of a single melatonin dose (2.5 mg) is to reduce rectal temperature during intermittent exercise in a hot environment without any alertness [193]. Independently from physical training, Liu et al. reported that melatonin (20 mg/kg) intravenously administered for four weeks enhanced lipolysis in mice *vastus lateralis* muscle by activating the “browning” effect in the adipose tissue and thermogenesis [194]. 

Remarkably, a recent comprehensive review set the point on the urgency to fill the existing gap on the best therapeutic melatonin dosage in human diseases despite a a great deal of experience in rodents [195]. Currently, melatonin is not still administered at the best “clinical” dosage, from 40 to 100 mg/day, that is necessary to obtain the best results in metabolic and neurodegenerative diseases. This “therapeutic range” is defined by the human equivalent dose (HED) considering a 75 kg adult men and normalization of body surface area [196]

Melatonin schedule treatment for skeletal muscle damage or activity is summarized in Table 2.

## 5. The Emerging Concept of the Gut–Muscle Axis—Role of Exercise and Melatonin in the Gut

Within the past few years, a novel and intriguing concept has been formulated: muscle composition and metabolism greatly depend on the bacteria population in the gut, called the microbiome. Thus, factors affecting the inter-individual microbiome such as aging, metabolic diseases, inflammation, cancer, or malnutrition might condition muscle weakness and induce sarcopenia [197,198,199]. Indeed, in age related diseases and obesity, an abnormal intestinal flora, i.e., dysbiosis, was detected [200,201,202]. The analysis of the composition of the intestinal microbiota in the elderly demonstrated that there were more pathogens such as *Enterobacteriaceae* and scarce butyrate-producing healthy bacteria, leading to less tight junctions in the mucosa and altered permeability [203]. 

Bindels and Delzenne suggested that the gut bacteria might be a potential therapeutic target to change muscle mass in cancer cachexia or undernutrition and hypothesized gut–muscle axis [204]. Firstly, Backhed et al. transplanted fecal bacteria from the adult pathogen-free mice to germ-free mice, in the latter modifying the fat deposition in the liver and the metabolism [205]. This process is defined as “conventionalization” and indicates the implantation and the colonization of bacteria from the distal intestine of a lean donor in the gut of a recipient. In another study, the above authors reported that, in the *gastrocnemius* muscle of germ free mice lacking the anabolic fasting-induced adipose factor (Fiaf) and fed a Western diet, there was reduced fatty acid oxidation [206]. On the contrary, germ-free mice were resistant to a Western diet if compared to control pathogen-free mice with intact gut bacteria. A crucial study by Yan et al. demonstrated that germ-free mice receiving fecal microbiota from obese Rongchang pigs presented a similar fatty phenotype with higher intramuscular triglycerides. In particular, the *gastrocnemius* muscle of recipient mice presented altered myofibers composition with more type I slow-contracting versus type II B fast fibers [207]. The study firstly indicated that there was a direct influence of gut bacteria on skeletal muscle organization and metabolism in mice. Recently, Lahiri et al. analyzed plasma and muscles in germ-free mice without any microbiota and fed either a regular diet or a mix of single chain fatty acids (SCFAs) able to promote beneficial insulin sensitivity and mitochondrial biogenesis [208]. In the first experimental set, mice presented muscle atrophy and enhanced molecular signaling of atrophy. In the second experimental set, SCFAs produced by the gut ameliorated muscle strength and composition. Finally, when germ-free mice were transplanted with a fecal content of pathogen-free mice treated with antibiotics, they developed muscle atrophy and reduced function due to a reduced healthy microbiota. These data agreed with Manickam et al.’s study, which demonstrated dysbiosis in pathogen-free mice treated with the antibiotic metronidazole producing scarce *gastrocnemius* muscle mass, abnormal activation of atrophy genes, and insulin resistence [209]. Conversely, fecal transplantation of old human subjects with high physical performance, called high functioning, in germ-free mice induced a peculiar microflora rich in *Prevotella* and *Barnesiella* species and developed more grip strength [210]. 

However, if several studies in animals indicate the possibility of a gut–muscle axis, it is important to outline that the reverse signaling from the muscle to the gut might also occur. Indeed, microbes develop a symbiontic reaction with their host and, consequently, reduced physical activity in sarcopenic subjects may be associated with different microbiota composition and metabolism [211,212]. This last point might be considered when the physical performance is measured in humans by gait speed test, because gut bacteria synthesize neurotransmitters such as serotonin, norepinephrine, GABA, or dopamine are able to influence the neurological control of movement [213]. 

Intriguingly, exercise strongly influences microbiota composition and produces beneficial metabolites in rodents and men. Remarkably, evidence in germ-free mice documents reduced inflammation and best swim time when colonized with *Eubacterium* or *Clostridium* species or highest treadmill run time if colonized by *Veilonella* [214,215,216]. Scheiman et al. reported that, in marathon runners after exercise, the high microbiota content with *Veilonella* transplanted in mice produced an intense treadmill activity [217]. The main metabolite produced by *Veilonella* in the colon was lactate then converted into propionate and used to produce short fatty chain acids (SCFAs), such as n-butyrate, acetate, and propionate, a source of energy for physical performance. 

The balance between human microbiota *Bacteroidetes* or *Firmicutes* phyla in aerobic exercise is essential for health, and disrupted equilibrium of gut bacteria colonization may induce inflammation and metabolic and neurological diseases [218]. On the contrary, aerobic exercise influences *Firmicutes* growth, modulating via the microbiome–gut–brain axis the symptoms of irritable bowl syndrome, axiety, and depression [219,220]. 

However, if the exercise intensity is too strong, the gut microbiota composition is dysregulated, and overtrained Kunming mice presented inflammation, inducing *Helicobacter pylori* and increased risk of peptic ulcer [221]. Moreover, depletion of gut microbiota in mice treated with antibiotics for 21 days induced low running endurance and less fatigue in the *extensorum digitorum longus* muscle. This last result is related to reduced glycogen production by a scarce number of bacteria present in the gut that directly synthesize glucose [222]. 

Unfortunately, dysbiosis and low butyrate production in the gut decreased melatonin secretion and enhanced permeability and inflammation in several organs [223]. Paulose et al. detected in the human gastrointestinal system that *Enterobacter aerogenes* is sensitive to melatonin and is synchronously regulated in the daily swarming [224]. The authors hypothesized that melatonin might act as a local *Zeitgeber* in the gut. Moreover, there is evidence that butyrate supplementation stimulated melatonin secretion in the duodenal tissue and in human colon carcinoma Caco 2 cells [225]. 

Firstly, Xu et al. reported that melatonin (50 mg/kg) delivered by gavage was effective as a probiotic compound ameliorating gut dysbiosis in high fat fed mice [226]. Moreover, in obese mice, melatonin changed the proportion of *Firmicutes* to *Bacterioides* and enhanced *Akkermasia* species that restored intestinal barriers in obese mice. Another study reported that melatonin supplementation in drinking water increased the microbiota variability in obese mice [227]. However, microbiota from obese mice plus melatonin transplanted in antibiotic-treated high fat fed mice failed to ameliorate lipid metabolism [228]. 

Probiotic nutrient intake is beneficial for the gut–microbiome–muscle axis, and this is particularly evident during exercise. Indeed, the analysis of gut microbiome in athletes gives the best information on the general health status, and proper nutrition may influence its composition to obtain the best physical performance [229]. Several studies on men after strenuous exercise and athletes demonstrated that probiotics intake changed microbiota, limited “leaky gut”, reduced inflammatory cytokines, and potentiated muscle strength [230]. 

Moreover, a diet rich in protein directly regulated muscle mass, but peculiar protein composition is crucial to limit sarcopenia in aging [231]. Among the dietary proteins that best sustain muscle mass, whey protein is effective combined with resistance training to modulate gut microbiota and to prevent sarcopenia [232]. However, a strong inter-individual response to resistance training exists that is regulated by myogenic molecular circadian pathways [233]. Remarkably, the gut microbiota influenced anabolic resistance in the skeletal muscle in the elderly. A recent metabolomic study of fecal content demonstrated a good correspondence between bacteria in the gut and the visceral fat and obesity grade [234]. 

Finally, sleep deprived mice showed reduced gut microbiota and limited probiotic species such as *Bacteroides*, *Akkermasia,* and *Faecalibacterium* [235]. Interestingly, in this animal model, melatonin reversed abnormal microbiota composition, indicating that sleep deprivation might reduce local melatonin secretion and melatonin type-1 receptor activity in the gut. 

## 6. Conclusions and Perspectives

The reciprocal influence of aging, diet, exercise, and gut microbiome on skeletal muscle mass and strength requires urgent innovative research and clinical trials considering the progressive increase in the age of the population in the world. 

**Figure 2:**
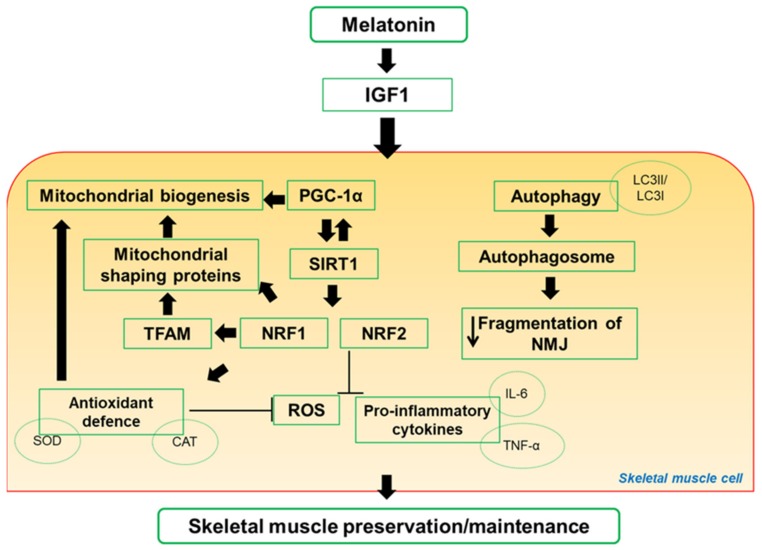
Scheme representation of proposal pathways regulated by melatonin in skeletal muscle. Notably, melatonin mechanisms of action involved mitochondria signaling. CAT: catalase; IL-6: interleukin-6; IGF1: insulin-like growth factor-1; LC3I/LC3II: microtubule-associated protein 1A/1B-light chain 3 free in the cytosol or conjugated to phosphotidylethanolamine during autophagy; NMJ: neuromuscular junction; NRF1 and NRF2: nuclear respiratory factor 1 and 2; PGC-1α: peroxisome proliferative activated receptor gamma coactivator-1alpha; ROS: reactive oxygen species; SIRT1: sirtuin1; SOD: superoxide dismutase; TFAM: mitochondrial transcription factor; TNFα: tumor necrosis factor alpha.

Melatonin is a highly evolutionary-conserved ancient molecule that was only recently rediscovered as a safe dietary supplement in muscle disorders and in exercise. This review attempts to shed light on potential and promising therapeutic roles of melatonin to limit muscle deterioration, mainly mitochondrial function, and sarcopenia. Main pathways activated by melatonin in skeletal muscle are drawn in Figure 2.

However, the utility of melatonin in athletes to obtain the best physical performance is strictly time-dependent, dose-dependent, and exercise-dependent. Finally, the benefit of melatonin on the gut microbiota is still very limited, and its direct influence on the gut–muscle axis is actually only speculative. Some limitations must be addressed in this manuscript. Firstly, we did not focus on the role of melatonin in cancer-induced cachexia due to the high number of existing reviews on cancer. Second, we did not consider differences in male compared to female or the role of sex hormones on muscle strength and exercise. Third, most of the studies on the gut–muscle axis were conducted on germ-free mice, not on humans. However, we are confident that new experimental studies and comphrensive reviews will be produced on these crucial themes in the future.

## Figures and Tables

**Table 1:** Main features of healthy and age-related skeletal muscle disorders in mammals.

	Healthy Middle-Aged	Age-Related Muscle Diseases	References
Myotubes size	Regular	Reduced	[48,50,57]
Satellite cells	Present	Reduced/Absent	[6,42,47]
Mitophagy	Normal	Aberrant	[55,59,60,65]
Neuromuscular junction	Regular	Absent	[9,10,12,41]
Triads/Calcium flux	Regular/Present	Disrupted/Absent	[26,27,49,74]
Mitochondria size/number	Regular Fission/Fusion	Megamitochondria Abnormal Fission/Fusion	[32,54,56,66]
Inflammation	Absent	Present	[3,52,62,70]
ROS formation	Absent/Minimal	High	[53,69,73]
ATP production	High	Limited	[61]
Microcirculation	Effective	Disrupted	[71,72,74]

*ROS, reactive oxygen species.

**Table 2:** Melatonin regime in skeletal muscle and exercise in rodents and humans.

Subjects/Cells	Dose	Times of Administration	Reference-Muscle Type or Exercise
Wistar albino rats	6 mg/kg s.c.	5 weeks	[86] *Soleus*
SAMP8 mice	10 mg/kg oral (water)	10 months	[87] *Diaphragm*
C57BL/6J mice	10 mg/kg oral (chow)	2 months	[90] *Gastrocnemius*
NLRP3 KO mice	10 mg/kg oral (chow)	2 months	[91] *Gastrocnemius*
Pinealectomized Wistar rats	0.5 mg/kg oral (water)	45 days	[92]
L6 cells	10 nM	24 h	[93]
C2C12	1–10 nM	20 min	[94]
C2C12 cells	100 mM	12–24 h	[95]
C2C12 cells	100 nM	16 h	[96]
Primary muscle cells	1–100 µM	24 h	[97]
Elderly patients	1 mg/day oral	4 weeks	[99]
Sprague-Dawley rats	10 mg/kg i.p.	30 min prior and immediately after reperfusion	[103] *Cremaster*
Sprague-Dawley rats	10 mg/kg i.v.	10 min prior and 10 min after reperfusion	[104] *Gracilis*
Wistar rats	10 mg/kg i.p.	4–14 days	[106] *Soleus*
Wistar rats	10 mg/kg i.p.	1–14 days	[107] *Soleus*
Wistar rats	20 mg/kg i.p.	7 days	[108]
Sprague-Dawley rats	2.5 mg/kg 5 mg/kg oral (water)	1–2 months	[110] *Gastrocnemius*
Sprague-Dawley rats	5 mg/kg oral (water)	2 months	[111] *Gastrocnemius*
Wistar rats	1 mg/kg oral (water)	16 weeks	[177] *Treadmill running*
Adult men	15 mg oral	Before starting exercise	[178] *High intensity run*
Wistar rats	20 mg/kg i.p.	Immediately after or 2 h after exercise	[179] *Treadmill running*
Adult subjects	6 mg oral	Before starting exercise	[180] *30 min graded exercise*
Football players	5 mg oral	30 days	[181] *Preparatory training*
Professionalsoccer players	6 mg oral	6 days	[182] *Intensive training*
Adult athletes	100 mg oral	4 weeks	[183] *Resistance training*
Adult athletes	20 mg oral	2 weeks	[184] *High Intensity training*
Wistar rat	10 mg/kg i.p.	2 days after exercise	[186] *Incremental swimming*
Teenage athletes	10 mg oral	After exercise	[192] *Exhaustive exercise*
Adult subjects	2.5 mg oral	Before exercise	[193] *Intermittent running*

## References

[1] Janssen I., Heymsfield S.B., Wang Z.M., Ross R. (1985). Skeletal muscle mass and distribution in 468 men and women aged 18-88 yr. J. Appl. Physiol..

[2] Frontera W.R., Ochala J. (2015). Skeletal muscle: A brief review of structure and function. Calcif. Tissue Int..

[3] Shadrin I., Khodabukus A., Bursac N. (2016). Striated muscle function, regeneration, and repair. Cell. Mol. Life Sci..

[4] Giudice J., Taylor J. (2017). Muscle as a paracrine and endocrine organ. Curr. Opin. Pharmacol..

[5] Roman W., Gomes E.R. (2018). Nuclear positioning in skeletal muscle. Semin. Cell Dev. Biol..

[6] Dumont N.A., Bentzinger C.F., Sincennes M.C., Rudnicki M.A. (2015). Satellite cells and skeletal muscle regeneration. Compr. Physiol..

[7] Gillies A.R., Bushong E.A., Deerinck T.J., Ellisman M.H., Lieber R.L. (2014). Three-dimensional reconstruction of skeletal muscle extracellular matrix ultrastructure. Microsc. Microanal..

[8] Hendrickse P., Degens H. (2019). The role of the microcirculation in muscle function and plasticity. J. Muscle Res. Cell Motil..

[9] Lepore E., Casola I., Dobrowolny G., Musaro’ A. (2019). Neuromuscolar junction as an entity of nerve-muscle communication. Cells.

[10] Slater C.R. (2017). The structure of human neuromuscular junctions. Some unanswered molecular questions. Int. J. Mol. Sci..

[11] Vock R., Weibel E., Hoppeler H., Ordway G., Weber J., Taylor C. (1996). Design of the oxygen and substrate supply to muscle cells. J. Exp. Biol..

[12] Boncompagni S., Rossi A., Micaroni M., Beznoussenko G., Polishchuk R., Dirksen R., Protasi F. (2009). Mitochondria are linked to calcium stores in striated muscle by developmentally regulated tethering structures. Mol. Biol. Cell..

[13] Ferreira R., Vitorino R., Alves R., Appel H., Powers S., Duarte J., Amado F. (2010). Subsarcolemmal and intermyofibrillar mitochondria proteome differences disclose functional specializations in skeletal muscles. Proteomics.

[14] Dahl R., Larsen S., Dohlmann T., Qvortrup K., Helge J., Dela F., Prats C. (2015). Three-dimensional reconstruction of the human skeletal muscle mitochondrial network as a tool to assess mitochondrial content and structural organization. Acta Physiol. (Oxf.).

[15] Bleck C., Kim Y., Willingham T., Glancy B. (2018). Subcellular connectomic analyses of energy networks in striated muscle. Nat. Commun..

[16] Barbieri E., Sestili P. (2012). Reactive oxygen species in skeletal muscle signaling. J. Signal. Transduct..

[17] Glancy B., Hartnell L., Malide D., Yu Z., Combs C., Connelly P., Subramaniam S., Balaban R. (2015). Mitochondrial reticulum for cellular energy distribution in muscle. Nature.

[18] Vincent A., White K., Davey T., Philips J., Ogden T., Lawless C., Warren C., Hall M., Ng Y., Falkous G. (2019). Quantitative 3D mapping of the human skeletal muscle mitochondrial network. Cell Rep..

[19] Liesa M., Shirihai O. (2013). Mitochondrial dynamics in the regulation of nutrient utilization and energy expenditure. Cell. Metab..

[20] Mishra P., Varuzhanyan G., Pham A., Chan D. (2015). Mitochondrial dynamics is a distinguishing feature of skeletal muscle fiber types and regulates organellar compartmentalization. Cell. Metab..

[21] Pette D., Staron R. (2000). Myosin isoforms, muscle fiber types, and transitions. Microsc. Res.Tech..

[22] Schiaffino S., Reggiani C. (2011). Fiber types in mammalian skeletal muscles. Physiol. Rev..

[23] Talbot J., Maves L. (2016). Skeletal muscle fiber type: Using insights from muscle developmental biology to dissect targets for susceptibility and resistance to muscle disease. WIREs Dev. Biol..

[24] Bourdeau J., Sephton C., Dutchak P. (2018). Metabolic networks influencing skeletal muscle fiber composition. Front. Cell Dev. Biol..

[25] Szent-Gyorgyi A. (2004). The early history of the biochemistry of muscle contraction. J. Gen. Physiol..

[26] Franzini-Armstrong C., Boncompagni S. (2011). The evolution of the mitochondria-to-calcium release units relationship in vertebrate skeletal muscles. J. Biomed. Biotechnol..

[27] Rossi A., Boncompagni S., Dirksen R. (2009). Sarcoplasmic reticulum-mitochondrial symbiosis: Bidirectional signaling in skeletal muscle. Exerc. Sport Sci. Rev..

[28] Ogata T., Yamasaki Y. (1997). Ultra-high resolution electron microscopy of mitochondria and sarcoplasmic reticulum arrangement in human red, white, and intermediate muscle fibers. Anat. Rec..

[29] Westerblad H., Bruton J., Katz A. (2010). Skeletal muscle: Energy metabolism, fiber types, fatigue and adaptability. Exp. Cell Res..

[30] Zierath J., Hawley J. (2004). Skeletal muscle fiber type: Influence on contractile and metabolic properties. PLoS Biol..

[31] Jeon Y., Choi J., Kim H., Lee H., Lim J., Choi S. (2019). Sex and fiber-type-related contractile properties in human single muscle fiber. J. Exerc. Rehabil..

[32] Hood D., Memme J., Oliveira A., Triolo M. (2019). Maintenance of skeletal muscle mitochondria in health, exercise, and Aging. Ann. Rev. Physiol..

[33] Mishra P., Chan D. (2016). Metabolic regulation of mitochondrial dynamics. J. Cell. Biol..

[34] Chen H., Chomyn A., Chan D. (2005). Disruption of fusion results in mitochondrial heterogeneity and dysfunction. J. Biol. Chem..

[35] Twig G., Shirihai O. (2011). The interplay between mitochondrial dynamics and mitophagy. Antioxid. Redox Signal..

[36] Gouspillou G., Bourdel-Marchasson I., Rouland R., Calmettes G., Biran M., Deschodt-Arsac V., Miraux S., Thiaudiere E., Pasdois P., Detaille D. (2014). Mitochondrial energetics is impaired in vivo aged skeletal muscle. Aging Cell.

[37] Picard M., Ritchie D., Wright K., Romestaing C., Thomas M., Rowan S., Taivassalo T., Hepple R. (2010). Mitochondrial functional impairment with aging is exaggerated in isolated mitochondria compared to permeabilized myofibers. Aging Cell.

[38] Choi S.J. (2016). Age-related functional changes and susceptibility to eccentric contraction-induced damage in skeletal muscle cell. Integr. Med. Res..

[39] Leduc-Gaudet J., Picard M., St-Jean Pelletier F., Sgarioto N., Auger M., Vallee J., Robitaille R., St-Pierre D., Gouspillou G. (2015). Mitochondrial morphology is altered in atrophied skeletal muscle of aged mice. Oncotarget.

[40] Delbono O. (2011). Expression and regulation of excitation-contraction coupling proteins in aging skeletal muscle. Curr. Aging Sci..

[41] Jang Y., Van Remmen H. (2011). Age-associated alterations of the neuromuscular junction. Exp. Gerontol..

[42] Miljkovic N., Lim J., Miljkovic I., Frontera W. (2015). Aging of skeletal muscle fibers. Ann. Rehab. Med..

[43] Cruz-Jentoft A., Bahat G., Bauer J., Boirie J., Bruyere O., Cederholm T., Cooper C., Landi F., Rolland Y., Sayer A. (2019). Sarcopenia: Revised European consensus on definition and diagnosis. Age Ageing.

[44] Carter H., Chen C., Hood D. (2015). Mitochondria, muscle health, and exercise with advancing age. Physiology.

[45] Calvani R., Joseph A., Adhihetty P., Miccheli A., Bossola M., Leeuwenburgh C., Bernabei R., Marzetti E. (2013). Mitochondrial pathways in sarcopenia of aging and disuse muscle atrophy. Biol. Chem..

[46] Fanzani A., Conraads V., Penna F., Martinet W. (2012). Molecular and cellular mechanisms of skeletal muscle atrophy: An update. J. Cachexia Sarcopenia Muscle.

[47] Hikida R. (2011). Aging changes in satellite cells and their functions. Curr. Aging Sci..

[48] Larsson L., Degens H., Li M., Salviati L., Lee Y., Thompson W., Kirkland J., Sandri M. (2019). Sarcopenia: Aging-related loss of muscle mass and function. Physiol. Rev..

[49] Zhao X., Weisleder N., Thornton A., Oppong Y., Campbell R., Ma J., Brotto M. (2008). Compromised store-operated Ca^2+^ entry in aged skeletal muscle. Aging Cell.

[50] Sayed R., de Leonardis E., Guerrero-Martinez J., Rahim I., Mokhtar D., Saleh A., Abdalla K., Pozo M., Escames G., López L. (2016). Identification of morphological markers of sarcopenia at early stage of aging in skeletal muscle of mice. Exp. Gerontol..

[51] Zhu S., Tian Z., Torigoe D., Zhao J., Xie P., Sugizaki T., Sato M., Horiguchi H., Terada K., Kadomatsu T. (2019). Aging-and obesity-related peri-muscular adipose tissue accelerates muscle atrophy. PLoS ONE.

[52] Fougere B., Boulanger E., Nourhashemi F., Guyonnet S., Cesari M. (2017). Chronic inflammation: Accelerator of biological aging. J. Gerontol. A Biol. Sci. Med. Sci..

[53] Marzetti E., Picca A., Marini F., Biancolillo A., Coelho Junior H., Gervasoni J., Bossola M., Cesari M., Onder G., Landi F. (2019). Inflammatory signatures in older persons with physical frailty and sarcopenia: The frialty “cytokinome” at its core. Exp. Gerontol..

[54] Szentesi P., Csernoch L., Dux L., Keller-Pinter A. (2019). Changes in Redox Signaling in the skeletal muscle with aging. Oxid. Med. Cell. Long..

[55] Del Campo A. (2019). Mitophagy as a new therapeutic target for sarcopenia. Acta Physiol..

[56] Sheard P., Anderson R. (2012). Age-related loss of muscle fibers is highly variable among mouse skeletal muscles. Biogerontology.

[57] Nilwik R., Snijders T., Leenders M., Groen B., van Kranenburg J., Verdijk L., van Loon L. (2013). The decline in skeletal muscle mass with aging is mainly attributed to a reduction in type II muscle fiber size. Exp. Gerontol..

[58] Del Campo A., Contreras-Hernandez I., Castro-Sepulveda M., Campos C., Figueroa R., Tevy M., Eisner V., Casas M., Jainovich E. (2018). Muscle function decline and mitochondria changes in middle age precede sarcopenia in mice. Aging.

[59] Pernas L., Scorrano L. (2018). Mito-Morphosis: Mitochondrial fusion, fission and cristae remodeling as key mediators of cellular function. Ann. Rev. Physiol..

[60] Romanello V., Sandri M. (2015). Mitochondrial quality control and muscle mass maintenance. Front. Physiol..

[61] Le Moal E., Pialoux V., Juban G., Groussard C., Zouhal H., Chazaud B., Mounier R. (2017). Redox control of skeletal muscle regeneration. Antiox. Redox Signal..

[62] Zhou R., Yazdi A., Menu P., Tschopp J. (2011). A role for mitochondria in NLRP3 inflammasome activation. Nature.

[63] Valentine J., Li M., Shoelson S., Zhang N., Reddick R., Musi N. (2018). NF-kB regulates muscle development and mitochondrial function. J. Gerontol. A Biol. Sci. Med. Sci..

[64] Johnson M., Robinson M., Nair K. (2013). Skeletal muscle aging and the mitochondrion. Trends Endocrinol. Metab..

[65] Yeo D., Kang C., Gomez-Cabrera M., Vina J., Ji L. (2019). Intensified mitophagy in skeletal muscle with aging is downregulated by PGC-1 alpha overexpression in vivo. Free Rad. Biol. Med..

[66] Huang D., Fan S., Chen X., Yan X., Zhang X., Ma B., Yu D., Xiao W., Zhuang C., Yu Z. (2019). Nrf2 deficiency exacerbates frailty and sarcopenia by impairing skeletal muscle mitochondrial biogenesis and dynamics in an age-dependent manner. Exp. Gerontol..

[67] Ciciliot S., Schiaffino S. (2010). Regeneration of mammalian skeletal muscle. Basic mechanisms and clinical implications. Curr. Pharm. Des..

[68] Abeles A., Pillinger M., Solitar B., Abeles M. (2007). Narrative review: The pathophysiology of fibromyalgia. Ann. Intern. Med..

[69] Chung C.P., Titova D., Oeser A., Randels M., Avalos I., Milne G.L., Morrow J.D., Stein C. (2009). Oxidative stress in fibromyalgia and its relationship to symptoms. Clin. Rheumatol..

[70] Cordero M.D., Alcocer-Gómez E., Culic O., Carrión A.M., de Miguel M., Díaz-Parrado E., Pérez-Villegas E.M., Bullón P., Battino M., Sánchez-Alcazar J.A. (2014). NLRP3 inflammasome is activated in fibromyalgia: The effect of coenzyme Q10. Antioxid. Redox Signal..

[71] Picard M., Hepple R., Burelle Y. (2012). Mitochondrial functional specialization in glycolytic and oxidative muscle fibers: Tailoring the organelle for optimal function. Am. J. Physiol. Cell Physiol..

[72] Charles A., Guilbert A., Guillot M., Talha S., Lejay A., Meyer A., Kindo M., Wolff V., Bouitbir J., Zoll J. (2017). Muscles susceptibility to ischemia-reperfusion injuries depends on fiber type specific antioxidant level. Front. Physiol..

[73] Guiraud S., Aartsma-Rus A., Vieira N., Davies K., Van Ommen G., Kunkel L. (2015). The pathogenesis and therapy of muscular dystrophies. Ann. Rev. Genom. Human Genet..

[74] Verhaart I., Aartsma-Rus A. (2019). Therapeutic developments for Duchenne muscular dystrophy. Nat. Rev. Neurol..

[75] Tan D., Hardeland R., Manchester L., Paredes S., Korkmaz A., Sainz R., Mayo J., Fuentes-Broto L., Reiter R. (2010). The changing biological roles of melatonin during evolution: From an antioxidant to signals of darkness, sexual selection and fitness. Biol. Rev. Camb. Philos. Soc..

[76] Reiter R., Tan D., Rosales C., Manchester L. (2013). The universal nature, unequal distribution and antioxidant functions of melatonin and its derivatives. Mini-Rev. Med. Chem..

[77] Paradies G., Paradies V., Ruggiero F., Petrosillo G. (2015). Protective role of melatonin in mitochondrial dysfunction and related disorders. Arch. Toxicol..

[78] Reiter R., Tan D., Rosales-Corral S., Galano A., Zhou X., Xu B. (2018). Mitochondria: Central organelles for melatonin’s antioxidant and anti-aging actions. Molecules.

[79] Reiter R., Tan D., Galano A. (2014). Melatonin: Exceeding Expectations. Physiology.

[80] Acuña-Castroviejo D., Escames G., Venegas C., Diaz-Casado M., Lima-Cabello E., Lopez L., Rosales-Corral S., Tan D., Reiter R. (2014). Extra-pineal melatonin: Sources, regulation, and potential functions. Cell. Mol. Life Sci..

[81] Meng X., Li Y., Li S., Zhou Y., Gan R., Xu D., Li H. (2017). Dietary sources and bioactivities of Melatonin. Nutrients.

[82] Arnao M., Hernandez-Ruiz J. (2018). The potential of Phytomelatonin as a nutraceutical. Molecules.

[83] Bubenik G., Konturek S. (2011). Melatonin and aging: Prospects for human treatment. J. Physiol. Pharmacol..

[84] Hardeland R. (2019). Aging, Melatonin, and the Pro-Inflammatory and Anti-Inflammatory Networks. Int. J. Mol. Sci..

[85] Lee J., Kim J., Lee D. (2014). Urine melatonin levels are inversely associated with sarcopenia in postmenopausal women. Menopause.

[86] Oner J., Oner H., Sahin Z. (2008). Melatonin is as effective as testosterone in the prevention of soleus muscle atrophy induced by castration in rats. Anat. Rec..

[87] Rodriguez M., Escames G., Lopez L., Garcia J., Ortiz F., Lopez A., Acuña-Castroviejo D. (2007). Melatonin administration prevents cardiac and diaphragmatic mitochondrial oxidative damage in senescence-accelerated mice. J. Endocrinol..

[88] Dardevet D., Remond D., Peyron M. (2012). Muscle wasting and resistance of muscle anabolism: The “anabolic threshold concept” for adapted nutritional strategies during sarcopenia. Sci. World J..

[89] McBride M., Foley K., D’Souza D., Li Y., Lau T., Hawke T., Schertzer J. (2017). The NLRP3 inflammasome contributes to sarcopenia and lower muscle glycolytic potential in old mice. Am. J. Physiol. Endocrinol. Metab..

[90] Sayed R., Fernandez-Ortiz M., Diaz-Casado M., Rusanova I., Rahim I., Escames G., Lopez L., Mokhtar D., Acuña-Castroviejo D. (2018). The protective effect of melatonin against age-associated, sarcopenia-dependent tubular aggregate formation, lactate depletion, and mitochondrial changes. J. Gerontol. A Biol. Sci. Med. Sci..

[91] Sayed R., Fernandez-Ortiz M., Diaz-Casado M., Aranda-Martinez P., Fernandez-Martinez J., Guerra-Librero A., Escames G., Lopez L., Alsaadawy R., Acuña-Castroviejo D. (2019). Lack of NLRP3 inflammasome activation reduces age-dependent sarcopenia and mitochondrial dysfunction, favoring the prophylactict effect of melatonin. J. Gerontol. A Biol. Sci. Med. Sci..

[92] Teodoro B., Baraldi F., Sampaio I., Bomfim L., Queiroz A., Passos M., Carneiro E., Alberici R., Amaral F., Cipolla-Neto J. (2014). Melatonin prevents mitochondria dysfunction and insulin resistance in rat skeletal muscle. J. Pineal Res..

[93] Favero G., Rodella L., Nardo L., Giugno L., Cocchi M., Borsani E., Reiter R., Rezzani R. (2015). A comparison of melatonin and α-lipoic acid in the induction of antioxidant defences in L6 rat skeletal muscle cells. AGE.

[94] Ha E., Yim S., Chung J., Yoon K., Kang I., Cho Y., Balk H. (2006). Melatonin stimulates glucose transport via insulin receptor substrate-1/phosphatidylinositol 3-kinase pathway in C2C12 murine skeletal muscle cells. J. Pineal Res..

[95] Salucci S., Battistelli M., Baldassarri V., Burini D., Falcieri E., Burattini S. (2017). Melatonin prevents mitochondrial dysfunctions and death in differentiated skeletal muscle cells. Microsc. Res. Tech..

[96] Quan X., Wang J., Liang C., Zheng H., Zhang L. (2015). Melatonin inhibits tunicamycin-induced endoplasmic reticulum stress and insulin resistance in skeletal muscle cells. Biochem. Biophys Res. Commun..

[97] Hibaoui Y., Roulet E., Ruegg U. (2009). Melatonin prevents oxidative stress-mediated mitochondrial permeability transition and death in skeletal muscle cells. J. Pineal Res..

[98] Coto-Montes A., Boga J., Tan D., Reiter R. (2016). Melatonin as a potential agent in the treatment of sarcopenia. Int. J. Mol. Sci..

[99] Rondanelli M., Peroni G., Gasparri C., Infantino V., Nichetti M., Cuzzoni G., Spadaccini D., Perna S. (2019). Is a combination of melatonin and amino acids useful to sarcopenic elderly patients? A randomized trial. Geriatrics.

[100] Romanello V., Scalabrin M., Albiero M., Blaauw B., Scorrano L., Sandri S. (2019). Inhibition of the fission machinery mitigates OPA1 impairment in adult skeletal muscles. Cells.

[101] Favaro G., Romanello V., Varanita T., Desbats M., Morbidoni V., Tezze C., Albiero M., Canato M., Gherardi G., De Stefani D. (2019). DRP1-mediated mitochondrial shape controls calcium homeostasis and muscle mass. Nature Communication.

[102] Messina A., Knight K., Dowsing B., Zhang B., Phan L., Hurley J., Morrison W., Stewart A. (2000). Localization of inducible nitric oxide synthase to mast cells during ischemia/reperfusion injury of skeletal muscle. Lab. Invest..

[103] Wang W., Fang X., Stephenson L., Baynosa R., Khiabani K., Zamboni W. (2005). Microcirculatory effects of melatonin in rat skeletal muscle after prolonged ischemia. J. Pineal. Res..

[104] Wang W., Fang X., Stephenson L., Zhang X., Khiabani K., Zamboni W. (2011). Melatonin attenuates I/R–induced mitochondrial dysfunction in skeletal muscle. J. Surg. Res..

[105] Qazi T., Duda G., Ort M., Perka C., Geissler S., Winkler T. (2019). Cell therapy to improve regeneration of skeletal muscle injuries. J. Cachexia Sarcopenia Muscle.

[106] Mehanna R., Soliman G., Hassaan P., Sharara G., Abdel-Moneim R. (2017). Protective role of melatonin on skeletal muscle injury in rats. Int. J. Clin. Exp. Med..

[107] Stratos I., Richter N., Li Z., Zechner D., Mittlemeier T., Vollmar B., Riotter R. (2012). Melatonin restores muscle regeneration and enhances muscle function after crush injury in rats. J. Pineal Res..

[108] Ostjen C., Sakuno Rosa C., Minuzzo Hartmann R., Goncalves Schemitt E., Raskopf Colares J., Possa Marroni N. (2019). Anti-inflammatory and antioxidant effect of melatonin on recovery from muscular trauma induced in rats. Exp. Mol. Pathol..

[109] Caumo W., Hidalgo M., Soyuza A., Torres I., Antunes L. (2019). Melatonin is a biomarker of circadian dysregulation and is correlated with major depression and fibromyalgia symptom severity. J. Pain Res..

[110] Favero G., Trapletti V., Bonomini F., Stacchiotti A., Lavazza A., Rodella L., Rezzani R. (2017). Oral supplementation of melatonin protects against fibromyalgia-related skeletal muscle alterations in reserpine-induced myalgia rats. Int. J. Mol. Sci..

[111] Favero G., Bonomini F., Franco C., Rezzani R. (2019). Mitochondrial dysfunction in skeletal muscle of a fibromyalgia model: The potential benefits of melatonin. Int. J. Mol. Sci..

[112] Woodman K., Coles C., Lamande’ S., White J. (2016). Nutraceuticals and their potential to treat Duchenne muscular dystrophy: Separating the credible from the conjecture. Nutrients.

[113] Hibaoui Y., Reutenauer-Patte J., Patthey-Vaudens O., Ruegg U., Dorchies O. (2011). Melatonin improves function of the dystrophic mdx^5cv^ mouse, a model for Duchenne muscular dystrophy. J. Pineal Res..

[114] Mc Cormick R., Vasilaki A. (2018). Age-related changes in skeletal muscle: Changes to life-style as a therapy. Biogerontology.

[115] Nilsson M., Tarnopolsky M. (2019). Mitochondria and Aging-The role of exercise as a countermeasure. Biology.

[116] Seo D., Lee S., Kim N., Ko K., Rhee D., Han J. (2016). Age-related changes in skeletal muscle mitochondria: The role of exercise. Int. Med. Res..

[117] Distefano G., Goodpaster B. (2018). Effects of exercise and aging on skeletal muscle. Cold Spring Harb. Perspect. Med..

[118] Balan E., Schwalm C., Naslain D., Nielsen H., Francaux M., Deldicque L. (2019). Regular endurance exercise promotes fission, mitophagy, and oxidative phosphorylation in human skeletal muscle independently of age. Front. Physiol..

[119] Laurin J., Reuid J., Lawrence M., Miller B. (2019). Long-term aerobic exercise preserves muscle mass and function with age. Curr. Opin. Physiol..

[120] Drake J., Yan Z. (2017). Mitophagy in maintaining skeletal muscle mitochondrial proteostasis and metabolic health with ageing. J. Physiol..

[121] Always S., Mohamed J., Myers M. (2017). Mitochondria initiate and regulate sarcopenia. Exerc. Sport Sci. Rev..

[122] Thompson L. (2002). Skeletal muscle adaptations with age, inactivity, and therapeutic exercise. J. Orthop. Sports Phys. Ther..

[123] Akasaki Y., Ouchi N., Izumiya Y., Bernardo B., Le Brasseur N., Walsh K. (2013). Glycolytic fast-twitch muscle fiber restoration counters adverse age-related changes in body composition and metabolism. Aging Cell.

[124] Jacobs R., Diaz V., Soldini L., Haider T., Thomassen M., Nordsborg N., Gassmann M., Lundby C. (2013). Fast-twitch glycolytic skeletal muscle is predisposed to age-induced impairments in mitochondrial function. J. Gerontol. A Biol. Sci. Med. Sci..

[125] Crupi A., Nunnelee J., Taylor D., Thomas A., Vit J., Riera C., Gottlieb R., Goodridge H. (2018). Oxidative muscles have better mitochondrial homeostasis than glycolytic muscles throughout life and maintain mitochondrial function during aging. Aging.

[126] Menshikova E., Ritov V., Fairfull L., Ferrell R., Kelley D., Goodpaster B. (2006). Effects of exercise on mitochondrial content and function in aging human skeletal muscle. J. Gerontol. Biol. Sci..

[127] Gan Z., Fu T., Kelly D., Vega R. (2018). Skeletal muscle mitochondrial remodeling in exercise and diseases. Cell Res..

[128] Coen P., Music R., Hinkley J., Miller B. (2019). Mitochondria as a target for mitigating sarcopenia. Front. Physiol..

[129] Ahmetov I., Vinogradova O., Williams A. (2012). Gene polymorphism and fiber-type composition of human skeletal muscle. Int. J. Sport Nutrit. Exerc. Metab..

[130] Flück M., Kramer D., Fitze D., Kasper S., Franchi M., Valdivieso P. (2019). Cellular aspects of muscle specialization demonstrate genotype-phenotype interaction effects in athletes. Front. Physiol..

[131] Valdivieso P., Vaughan D., Laczko E., Broglioli M., Waldron S., Rittweger J., Flück M. (2017). The metabolic response of skeletal muscle to endurance exercise is modified by the ACE-I/D gene polymorphism and training state. Front. Physiol..

[132] Chen C., Erlich A., Hood D. (2018). Role of Parkin and endurance training on mitochondrial turnover in skeletal muscle. Skelet. Muscle.

[133] Chen C., Erlich A., Crilly M., Hood D. (2018). Parkin is required for exercise-induced mitophagy in muscle: Impact of aging. Am. J. Physiol. Metab..

[134] Erlich A., Hood D. (2019). Mitophagy regulation in skeletal muscle: Effect of endurance exercise and age. J. Science Sport Exerc..

[135] Erlich A., Brownlee D., Beyfuss K., Hood D. (2018). Exercise induces TFEB expression and activity in skeletal muscle in a PGC-1α-dependent manner. Am. J. Physiol. Cell Physiol..

[136] Settembre C., Ballabio A. (2011). TFEB regulates autophagy: An integrated coordination of cellular degradation and recycling processes. Autophagy.

[137] Vainshtein A., Tryon L., Pauly M., Hood D. (2015). Role of PGC-1α during acute exercise-induced autophagy and mitophagy in skeletal muscle. Am. J. Physiol. Cell Physiol..

[138] Uguccioni G., Hood D. (2011). The importance of PGC-1α in contractile activity-induced mitochondrial adaptations. Am. J. Physiol. Endocrinol. Metab..

[139] Carter H., Kim Y., Erlich A., Zarrin-Khat D., Hood D. (2018). Autophagy and mitophagy flux in young and aged skeletal muscle following chronic contractile activity. J. Physiol..

[140] Triolo M., Hood D. (2019). Mitochondrial breakdown in skeletal muscle and the emerging role of the lysosomes. Arch. Biochem. Biophys..

[141] Preisler N., Laforêt P., Madsen K., Husu E., Vissing C., Hedermann G., Galbo H., Lindberg C., Vissing J. (2017). Skeletal muscle metabolism during prolonged exercise in Pompe disease. Endocrin. Connect..

[142] Van den Berg L., Favejee M., Wens S., Kruijshaar M., Praet S., Pijnappel W., van Doorn P., Bussmann J., van der Ploeg A. (2015). Exercise training in adults with Pompe disease: The effects on pain, fatigue, and functioning. Arch. Phys. Med. Rehabil..

[143] Mcleod J., Stokes T., Phillips S. (2019). Resistance exercise training as a primary countermeasure to age-related chronic disease. Front. Physiol..

[144] Piccirillo R. (2019). Exercise-induced myokines with therapeutic potential for muscle wasting. Front. Physiol..

[145] Standford K., Goodyear L. (2018). Muscle-adipose tissue cross talk. Cold Spring Harb. Perspect. Med..

[146] Tanimura Y., Aoi W., Takanami Y., Kawai Y., Mizushima K., Naito Y., Yoshikawa T. (2016). Acute exercise increases fibroblast growth factor 21 in metabolic organs and circulation. Physiol. Rep..

[147] Izumiya Y., Bina H., Ouchi N., Akasaki Y., Kharitonenkov A., Walsh K. (2008). FGF21 is an Akt-regulated myokine. FEBS Lett..

[148] Dong J., Dong Y., Dong Y., Chen F., Mitch W., Zhang L. (2016). Inhibition of myostatin in mice improves insulin sensitivity via irisin-mediated cross talk between muscle and adipose tissues. Int. J. Obes..

[149] Szuhany K., Bugatti M., Otto M. (2015). A meta-analytic review of the effects of exercise on brain-derived neurotrophic factor. J. Psychiatr. Res..

[150] He Z., Tian Y., Valenzuela P., Huang C., Zhao J., Hong P., He Z., Yin S., Lucia A. (2018). Myokine response to high-intensity interval vs resistance exercise: An individual approach. Front. Physiol..

[151] Safdar A., Saleem A., Tarnopolsky M. (2016). The potential of endurance exercise-derived exosomes to treat metabolic diseases. Nat. Rev. Endocrinol..

[152] Trovato E., Di Felice V., Barone R. (2019). Extracellular vesicles: Delivery vehicles of myokines. Front. Physiol..

[153] Vaughan S., Wallis M., Polit D., Steele M., Shum D., Morris N. (2014). The effects of multimodal exercise on cognitive and physical functioning and brain-derived neurotrophic factor in older women: A randomized controlled trial. Age and Ageing.

[154] Suire C., Eitan E., Shaffer N., Tian Q., Studenki S., Mattson M., Kapogiannis D. (2017). Walking speed decline in older adults is associated with elevated pro-BDNF in plasma extracellular vesicles. Exp. Gerontol..

[155] Hastings M., O’Neill J., Maywood E. (2007). Circadian clocks: Regulators of endocrine and metabolic rhythms. J. Endocrinol..

[156] De Goede P., Wefers J., Brombacher E., Schrauwen P., Kalsbeek A. (2018). Circadian rhythms in mitochondrial respiration. J. Mol. Endocrinol..

[157] Doherty R., Madigan S., Warrington G., Ellis J. (2019). Sleep and nutrition interactions: Implications for athletes. Nutrients.

[158] Welsh D., Takahashi J., Kay S. (2010). Suprachismatic nucleus: Cell autonomy and network properties. Annu. Rev. Physiol..

[159] Dibner C., Schibler U., Albrecht U. (2010). The mammalian circadian timing system: Organization and coordination of central and peripheral clocks. Annu. Rev. Physiol..

[160] Yanar K., Simsek B., Cakatay U. (2019). Integration of Melatonin related redox homeostasis, aging, and circadian rhythm. Rejuvenation Res..

[161] Nakamura T., Takasu N., Nakamura W. (2016). The suprachiasmatic nucleus: Age-related decline in biological rhythms. J. Physiol. Sci..

[162] Arendt J. (2019). Melatonin: Countering chaotic time cues. Front. Endocrinol..

[163] Kandalepas P., Mitchell J., Gillette M. (2016). Melatonin signal transduction pathways require E-Box-Mediated transcription of Per 1 and Per 2 to reset the SCN clock at dusk. PLoS ONE.

[164] Huang R. (2018). The discoveries of molecular mechanisms for the circadian rhythm: The 2017 Nobel Prize in physiology or medicine. Biomed. J..

[165] Harfmann B., Schroeder E., Esser K. (2015). Circadian rhythms, the molecular clock, and skeletal muscle. J. Biol. Rhythms.

[166] Chatterjee S., Ma K. (2016). Circadian clock regulation of skeletal muscle growth and repair. F1000 Res..

[167] Vitale J., Bonato M., La Torre A., Banfi G. (2019). The role of the molecular clock in promoting skeletal muscle growth and protecting against sarcopenia. Int. J. Mol. Sci..

[168] Nohara K., Mallampalli V., Nemkov T., Wirianto M., Yang J., Ye Y., Sun Y., Han L., Esser K., Mileykovskaya E. (2019). Nobiletin fortifies mitochondrial respiration in skeletal muscle to promote healthy aging against metabolic challenge. Nat. Commun..

[169] Schiaffino S., Reggiani C., Murgia M. (2019). Fiber type divesity in skeletal muscle explored by mass spectrometry-based single fiber proteomics. Histology Histopathol..

[170] Mirizio G., Nunes Mendes R., Castillo Figueroa A., de Sousa Junior I., Pimentel Ferreira A., Vieira E. (2018). The impact of physical exercise on the skeletal muscle clock genes. Kinesiology.

[171] Melancon M., Lorrain D., Dionnbe I. (2015). Sleep depth and continuity before and after chronic exercise in older man: Electrophysiological evidence. Physiol. Behav..

[172] Carlson l., Pobocik K., Lawrence M., Brazeau D., Koch A. (2019). Influence of exercise time of day on salivary melatonin responses. Int. J. Sports Physiol. Perf..

[173] Escames G., Ozturk G., Baño-Otalora B., Pozo M., Madrid J., Reiter R., Serrano E., Concepcion M., Acuña-Castroviejo D. (2012). Exercise and melatonin in humans: Reciprocal benefits. J. Pineal Res..

[174] Obayashi K., Saeki K., Maegawa T., Iwamoto J., Sakai T., Otaki N., Kataoka H., Kurumatani N. (2016). Melatonin secretion and muscle strength in elderly individuals: A cross-sectional study of the HEIJO-KYO Cohort. J. Gerontol. A Biol. Med. Sci..

[175] Thrift A., Xiao L., Patel S., Tworoger S., Mc Tiernan A., Duggan C. (2014). Effects of physical activity on melatonin levels in previously sedentary men and women. Cancer Epidemiol. Biomarkers Prev..

[176] De Aquino L., dos Santos Thomatieli R., Antunes Moreira H., Behn C., Viscor G., Lira Santos F., Bittar I., Caris Venticinque A., Tufik S., De Mello M. (2018). Melatonin and sleep responses to normobaric hypoxia and aerobic physical exercise: A randomized controlled trial. Physiol. Behav..

[177] Mendes C., Lopes Sousa A., Do Amaral Gaspar F., Peliciari-Garcia R., de Oliveira Turati A., Hirabara S., Scialfa F., Cipolla-Neto J. (2013). Adaptations of the aging animal to exercise: Role of daily supplementation with melatonin. J. Pineal Res..

[178] Ochoa J., Diaz-Castro J., Kajarabille N., Garcia C., Guisado I., De Teresa C., Guisado R. (2011). Melatonin supplementation ameliorates oxidative stress and inflammatory signaling induced by strenuous exercise in adult human males. J. Pineal Res..

[179] Borges da Silva L., Dermargos A., Pinto da Silva Junior E., Weimann E., Herling Lambertucci R., Hatanaka E. (2015). Melatonin decreases muscular oxidative stress and inflammation induced by strenuous exercise and stimulates growth factor synthesis. J. Pineal Res..

[180] Trionfante C., Davis G., Farney T., Miskowiec R., Nelson A. (2017). A pre-exercise dose of melatonin can alter substrate use during exercise. Int. J. Exer. Sci..

[181] Czuczejko J., Sielski L., Wozniak B., Wozniak A., Szewczyk-Golec K. (2019). Melatonin supplementation improves oxidative and inflammatory state in the blood of professional athletes during the preparatory period for competitions. Free Rad. Res..

[182] Farjallah M., Hammouda O., Mahmoud L., Graja A., Ghattassi K., Boudaya M., Jammoussi K., Sahnoun Z., Souissi N. (2018). Melatonin supplementation ameliorates oxidative stress, antioxidant status and physical performances recovery during soccer training camp. Biol. Rhythm Res..

[183] Leonardo-Mendonça R., Ocaña-Wilhelmi J., de Haro T., de Teresa-Galvan C., Guerra-Hernandez E., Rusanova I., Fernandez-Ortiz M., Sayed R., Escames G., Acuña-Castroviejo D. (2017). The benefit of a supplement with the antioxidant melatonin on redox status and muscle damage in resistance-trained athletes. Appl. Physiol. Nutr. Metab..

[184] Ortiz-Franco M., Planells E., Quintero B., Acuña-Castroviejo D., Rusanova I., Escames G., Molina-Lopez J. (2017). Effect of melatonin supplementation on antioxidant status and DNA damage in high intensity trained athletes. Int. J. Sport Med..

[185] Maarman G., Reiter R. (2018). Melatonin therapy for blunt trauma and strenuous exercise: A mechanism involving cytokines, NFkB, Akt, MAF_BX_ and MURF-1. J. Sport Sci..

[186] Beck W., Botezelli J., Pauli J., Rochete Ropelle E., Gobatto C. (2015). Melatonin has an ergogenic effect but does not prevent inflammation and damage in exhaustive exercise. Sci. Rep..

[187] Beck W., Scariot P., Gobatto C. (2016). Melatonin is an ergogenic aid for exhaustive aerobic exercise only during the wakefulness period. Int. J. Sport Med..

[188] Andersen L., Gogenur I., Rosenberg J., Reiter R. (2016). The safety of melatonin in humans. Clin. Drug Invest..

[189] Chazaud B. (2016). Inflammation during skeletal muscle regeneration and tissue remodeling: Application to exercise-induced muscle damage management. Immunol. Cell Biol..

[190] Lopez-Flores M., Luque-Nieto R., Costa Moreira O., Suarez-Iglesias D., Villa-Vicente J. (2018). Effects of melatonin on sports performance: A systematic review. JEP Online.

[191] Roach G., Sargent C. (2019). Interventions to minimize jet lag after westward and eastward flight. Front. Physiol..

[192] Cheikh M., Hammouda O., Gaamouri N., Driss T., Chamari K., Cheikh R., Dogui M., Souissi N. (2018). Melatonin ingestion after exhaustive late-evening exercise improves sleep quality and quantity, and short-term performances in teenage athletes. Chronobiol. Int..

[193] Atkinson G., Holder A., Robertson C., Gant N., Drust B., Reilly T., Waterhouse J. (2005). Effects of melatonin on the thermoregulatory responses to intermittent exercise. J. Pineal Res..

[194] Liu K., Yu W., Wei W., Zhang X., Tian Y., Sherif M., Liu X., Dong C., Wu W., Zhang L. (2019). Melatonin reduces intramuscular fat deposition by promoting lipolysis and increasing mitochondrial function. J. Lipid Res..

[195] Cardinali D. (2019). Are melatonin doses employed clinically adequate for melatonin-induced cytoprotection?. Melatonin Res..

[196] Reagan-Shaw S., Nihal M., Ahmad N. (2008). Dose translation from animal to human studies revisited. FASEB J..

[197] Ticinesi A., Lauretani F., Milani C., Nouvenne A., Tana C., Del Rio D., Maggio M., Ventura M., Meschi T. (2017). Aging gut microbiota at the cross-road between nutrition, physical frailty, and sarcopenia: Is there a gut-muscle axis?. Nutrients.

[198] Grosicki G., Fielding R., Lustgarten M. (2018). Gut microbiota contribute to age-related changes in skeletal muscle size, composition, and function: Biological basis for gut-muscle axis. Calcif. Tissue Int..

[199] Genton L., Mareschal J., Charretier Y., Lazarevic V., Binndels L., Schrenzel J. (2019). Targeting the gut microbiota to treat cachexia. Front. Cell. Infect. Microbiol..

[200] Lustgarten M. (2019). The role of the gut microbiome on skeletal muscle mass and physical function: 2019 Update. Front. Physiol..

[201] Heintz C., Mair W. (2014). You are what you host: Microbiome modulation of the aging process. Cell.

[202] Picca A., Fanelli F., Calvani R., Mule’ G., Pesce V., Sisto A., Pantanelli C., Bernabei R., Landi F., Marzetti E. (2018). Gut dysbiosis and muscle aging: Searching for novel targets against sarcopenia. Mediat. Inflamm..

[203] Henao-Mejia J., Elinav E., Jin C., Hao L., Mehal W., Strowig T., Thaiss C., Kau A., Eisenbarth S., Jurczak M. (2012). Inflammasome-mediated dysbiosis regulates progression of NAFLD and obesity. Nature.

[204] Jeffery I., Lynch D., O’Toole P. (2016). Composition and temporal stability of the gut microbiota in older persons. ISME J..

[205] Bindels L., Delzenne N. (2013). Muscle wasting: The gut microbiota a a new therapeutic target?. J. Biochem. Cell Biol..

[206] Backhed F., Ding H., Wang T., Hooper L., Koh G., Nagy A., Semenkovich C., Gordon J. (2004). The gut microbiota as an environmental factor that regulates fat storage. PNAS.

[207] Backhed F., Manchester J., Semenkovich C., Gordon J. (2007). Mechanisms underlying the resistance to diet-induced obesity in germ-free mice. PNAS.

[208] Yan H., Diao H., Xiao Y., Wenxia L., Yu B., He J., Yu J., Zheng P., Mao X., Luo Y. (2016). Gut microbiota can transfer fiber characteristics and lipid metabolic profiles of skeletal muscle from pigs to germ-free mice. Sci. Rep..

[209] Lahiri S., Kim H., Garcia-Perez I., Rza M., Martin K., Kundu P., Cox L., Selkrig J., Posma J., Zhang H. (2019). The gut microbiota influences skeletal muscle mass and function in mice. Sci. Transl. Med..

[210] Manickam R., Oh H., Tan C., Paramalingam E., Wahli W. (2018). Metronidazole causes skeletal muscle atrophy and modulates muscle chronometabolism. Int. J. Mol. Sci..

[211] Fielding R., Reeves A., Jasuja R., Liu C., Barrett B., Lustgarten M. (2019). Muscle strenght is increased in mice that are colonized with microbiota from high-functioning older adults. Exp. Gerontol..

[212] Ticinesi A., Nouvenne A., Cerundolo N., Catania P., Prati B., Tana C., Meschi T. (2019). Gut microbiota, muscle mass and function in aging: A focus on physical frailty and sarcopenia. Nutrients.

[213] Mailing L., Allen J., Buford T., Fields C., Woods J. (2019). Exercise and the gut microbiome: A review of the evidence, potential mechanisms, and implications for human health. Exerc. Sport Sci. Rev..

[214] Strandwitz P. (2018). Neurotransmitter modulation by the gut microbiota. Brain Res..

[215] Huang W., Chen Y., Chuang H., Chiu C., Huang C. (2019). Investigation of the effects of microbiota on exercise physiological adaptation, performance, and energy utilization using a Gnotobiotic animal model. Front. Microbiol..

[216] Monda V., Villano I., Messina A., Valenzano A., Esposito T., Moscatelli F., Viggiano A., Cibelli G., Chieffi S., Monda M. (2017). Exercise modifies the gut microbiota with positive health effects. Oxid. Med. Cell. Long..

[217] Scheiman J., Luber J., Chavkin T., MacDonald T., Tung A., Pham L., Wibowo M., Wurth R., Punthambaker S., Tierney B. (2019). Meta-omics analysis of elite athletes identifies a performance-enhancing microbe that functions via lactate metabolism. Nat. Med..

[218] Allen J., Mailing L., Niemiro G., Moore R., Cook M., White B., Holscher H., Wood J. (2018). Exercise alters gut microbiota composition and function in lean and obese humans. Med. Sci. Sports Exerc..

[219] Zhao X., Zhang Z., Hu B., Huang W., Yuan C., Zou L. (2018). Response of gut microbiota to metabolite changes induced by endurance exercise. Front. Microbiol..

[220] Dalton A., Mermier C., Zuhl M. (2019). Exercise influence on the microbiome-gut-brain axis. Gut Microbes.

[221] Yuan X., Xu S., Huang H., Liang J., Wu Y., Li C., Yuan H., Zhao X., Lai X., Hou S. (2018). Influence of excessive exercise on immunity, metabolism, and gut microbial diversity in an overtraining mice model. Scand. J. Med. Sci. Sports.

[222] Nay K., Jollet M., Goustard B., Baati N., Vernus B., Pontones M., Lefeuvre-Orfila L., Bendavid C., Ruè O., Mariadassou M. (2019). Gut bacteria are critical for optimal muscle function: A potential link with glucose homeostasis. Am. J. Physiol. Endocrinol. Metab.

[223] Anderson G. (2019). Gut dysbiosis dysregulates central and systemic homeostasis via decreased melatonin and suboptimal mitochondria functioning: Pathoetiological and pathophysiological implications. Melat. Res..

[224] Paulose J., Wright J., Patel A., Cassone V. (2016). Human gut bacteria are sensitive to melatonin and express endogenous circadiam rhythmicity. PLoS ONE.

[225] Jin C., Engstler A., Sellmann C., Ziegenhardt D., Landmann M., Kanuri G., Lounis H., Schröder M., Vetter W., Bergheim I. (2016). Sodium butyrate protects mice from the development of the early signs of non-alcoholic fatty liver disease: Role of melatonin and lipid peroxidation. Br. J. Nutr..

[226] Xu P., Wang J., Hong F., Wang S., Jin X., Xue T., Jia L., Zhai Y. (2017). Melatonin prevents obesity through modulatrion of gut microbiota in mice. J. Pineal Res..

[227] Yin J., Li Y., Han H., Chen S., Gao J., Liu G., Wu X., Deng J., Yu Q., Huang X. (2018). Melatonin reprogramming of gut microbiota improves lipid dysmetabolism in high-fat diet-fed mice. J. Pineal Res..

[228] Mach N., Fuster-Botella D. (2017). Endurance exercise and gut microbiota: A review. J. Sports Health Sci..

[229] Lamprecht M., Bogner S., Schippinger G., Steinbauer K., Fankhauser F., Hallstroem S., Schuetz B., Greilberger J. (2012). Probiotic supplementation affects markers of intestinal barrier, oxidation, and inflammation in trained men; a randomized, double-blinded, placebo-controlled trial. J. Int. Soc. Sports Nutr..

[230] Wosinska L., Cotter P., O’Sullivan O., Guiname C. (2019). The potential impact of probiotics on the gut microbiome of athletes. Nutrients.

[231] Lochlainn M., Bowyer R., Steves C. (2018). Dietary protein and muscle in aging people: The potential role of the gut microbiome. Nutrients.

[232] Liao Y., Peng Z., Chen L., Zhang Y., Cheng Q., Nüssler A., Bao W., Liu L., Yang W. (2019). Prospective views for whey protein and/or resistance training against age-related sarcopenia. Aging Dis..

[233] Camera D. (2018). Anabolic heterogeneity following resistance training: A role for circadian rhythm?. Front. Physiol..

[234] Zierer J., Jackson M., Kastenmuller G., Mangino M., Long T., Telenti A., Mohney R., Small K., Bell J., Steves C. (2018). The fecal metabolome as a functional readout of the gut microbiome. Nat. Genet..

[235] Gao T., Wang Z., Dong Y., Cao J., Lin R., Wang X., Yu Z., Chen Y. (2019). Role of melatonin in sleep deprivation-induced intestinal barrier dysfunction in mice. J. Pineal Res..

